# Home administration of maintenance pemetrexed for patients with advanced non-squamous non-small cell lung cancer: rationale, practicalities and phase II feasibility study design

**DOI:** 10.1186/1477-7525-11-163

**Published:** 2013-10-03

**Authors:** Rohit Lal, Nawel Bourayou, Gunnar Hillerdal, Marianne Nicolson, Anders Vikstrom, Maria Lorenzo, Yulia D’yachkova, Susana Barriga, Carla Visseren-Grul

**Affiliations:** 1 Guy’s and St Thomas’ Foundation Trust, London, UK; 2 Eli Lilly, Neuilly sur Seine, France; 3 Karolinska Universitetsjukhuset, Solna, Sweden; 4 Aberdeen Royal Infirmary, Aberdeen, UK; 5 Universitetssjukhuset, Linkoping, Sweden; 6 Eli Lilly, Windlesham, Surrey, UK; 7 Eli Lilly GmbH, Vienna, Austria; 8 Eli Lilly, Madrid, Spain; 9 Eli Lilly, Houten, The Netherlands

**Keywords:** Pemetrexed, Lung, Maintenance treatment, Home administration

## Abstract

**Background:**

Home-based care in oncology is mainly reserved for patients at the end of life. Regulations regarding home delivery of cytotoxics differ across Europe, with a notable lack of practice guidelines in most countries. This has led to a lack of data addressing the feasibility of home-based administration of cytotoxic chemotherapy. In advanced non-squamous non-small cell lung cancer, pemetrexed is approved as maintenance therapy after first-line chemotherapy. In this setting, patients have the potential to be treated long-term with maintenance therapy, which, in the absence of unacceptable toxicity, is continued until disease progression. The favourable safety profile of pemetrexed and the ease of its administration by 10-minute intravenous infusion every 3 weeks make this drug a suitable candidate for administration in a home setting.

**Methods:**

Literature and regulations relevant to the home-based delivery of cytotoxic therapy were reviewed, and a phase II feasibility study of home administration of pemetrexed maintenance therapy was designed. At least 50 patients with advanced non-squamous non-small cell lung cancer, Eastern Cooperative Oncology Group performance status 0–1 and no progressive disease after four cycles of platinum-based first-line therapy are required to allow investigation of the feasibility of home-based administration of pemetrexed maintenance therapy (500 mg/m^2^ every 3 weeks until progressive disease or unacceptable toxicity). Feasibility is being assessed as adherence to the home-based administration process (primary endpoint), patient safety, impact on patients’ quality of life, patient and physician satisfaction with home care, and healthcare resource use and costs. Enrolment of patients from the UK and Sweden, where home-based care is relatively well developed, commenced in December 2011.

**Discussion:**

This feasibility study addresses an important aspect of maintenance therapy, that is, patient comfort during protracted home-based chemotherapy. The study design requires unusual methodology and specific logistics to address outcomes relevant to the home-delivery approach. This article presents a study design that offers a novel and reproducible model for home-based chemotherapy, and provides an up-to-date overview of the literature regarding this type of treatment.

**Trial registration:**

ClinicalTrials.gov: NCT01473563

## Background

Lung cancer is the most common cause of cancer-related death in Europe [1]. Non-small cell lung cancer (NSCLC) accounts for 80–85% of all lung cancer cases, and the majority of patients present with advanced disease [2].

Current guidelines for the treatment of patients with advanced NSCLC recommend platinum-based combination regimens using a third-generation agent as first-line treatment [3-5]. For patients who have not progressed at completion of first-line treatment and have good performance status (PS; 0–1), maintenance therapy is now recommended [3,4,6]. The aim of maintenance therapy is to delay disease progression and improve patient survival while preserving health-related quality of life (HRQoL) [7].

Because maintenance therapy is, by definition, long-term (as it relates to treatment until progression), optimising the experience of patients during therapy is important. In many countries, there has been an increasing trend of moving healthcare services, including those for cancer, into the community and into ambulatory or home-based care. Such moves aim to improve the choice and experience of patients, and make more effective and efficient use of resources [8]. In general, home-based treatment is associated with good patient satisfaction, acceptability and safety, and improved HRQoL [9-14]. Home-based administration could also increase access to treatment, reduce the need for patients to travel to hospital and wait for treatment, and increase time spent at home, all of which are important to patients and their families [9]. However, some down sides to home-based chemotherapy have been reported; for example, a lack of support from others in a similar position and feeling less secure at home [9,13]. In addition, it is possible that such an approach to therapy could increase the burden on relatives.

Home-based care in oncology is mainly reserved for patients at the end of life. Regulations regarding home administration of cytotoxics differ widely across Europe, with a notable lack of practice guidelines in most countries. This has led to an important lack of data regarding the feasibility of home-based administration of cytotoxic chemotherapy in patients with NSCLC. We identified one study in literature searches that considered home administration of cytotoxic chemotherapy in this patient population; this was a feasibility study that investigated the home-based use of gemcitabine monotherapy as first-line treatment in 24 patients with advanced NSCLC [9]. Patients and caregivers in this study reported positively on home-based administration and preferred it to hospital-based administration, with only one patient requiring a change from home- to hospital-based administration (because of feelings of anxiety and fainting after the second injection of the second cycle).

Pemetrexed, a multi-targeted antifolate that inhibits several enzymes of the folate pathway, is approved as a first-line (in combination with cisplatin) [15] and second-line (as a single agent) treatment of advanced non-squamous NSCLC [16]. Pemetrexed is also indicated as maintenance therapy for patients with advanced non-squamous NSCLC whose disease has not progressed after four cycles of platinum-based doublet induction chemotherapy and who have a good PS (Eastern Cooperative Oncology Group [ECOG] PS 0–1). This approval was based on the results of two phase III, randomi*s*ed, double-blind, placebo-controlled trials showing that single-agent pemetrexed (with best supportive care) maintenance therapy improved progression-free survival and overall survival (OS) [17,18] in this patient group. Patients who enrolled in these studies received long-term pemetrexed treatment (up to 55 cycles): in the study by Ciuleanu et al. [17], patients received a median of 5 cycles, with 48% (n = 213) and 23% of patients (n = 103) completing >6 cycles and ≥10 cycles of therapy, respectively, whereas in the PARAMOUNT study [18], patients received a median of four cycles, with 37% of patients (n = 133) and 28% (n = 99) completing >6 cycles and ≥10 cycles of therapy, respectively.

The efficacy of pemetrexed as maintenance therapy and its favourable and well-characterised safety profile, together with the ease of its administration by 10-minute intravenous (i.v.) infusion every 3 weeks, make this drug a suitable candidate for administration in an outpatient, home-based setting. Therefore, we initiated a study to investigate the feasibility of home-based administration of pemetrexed as maintenance therapy in patients with advanced non-squamous NSCLC. This article describes the study design and methodology, and discusses the practicalities of providing home-based administration of maintenance chemotherapy with pemetrexed to patients with advanced non-squamous NSCLC.

## Methods

### Study design and objectives

This is a multicentre, two-country, single-arm, open-label, phase II study, designed to evaluate the feasibility of administering pemetrexed as maintenance therapy in a home setting to patients with stage IIIB or IV non-squamous NSCLC.

The primary objective of this study is to assess the adherence rate to pemetrexed maintenance therapy administered in a home setting. Patients will be considered adherent unless they revert to infusions at hospital or discontinue from the study because of the home setting. Secondary objectives of the study include the assessment of resource utilisation, distances travelled, the number and length of visits, and unplanned use of healthcare resources (primary or emergency care, hospitalisations). Additional secondary objectives include the safety of pemetrexed administered in a home setting and characterisation of the types and incidences of drug toxicities; patient HRQoL; patient and physician satisfaction with home care; time-to-treatment failure (TTF); and OS.

**Figure 1:**
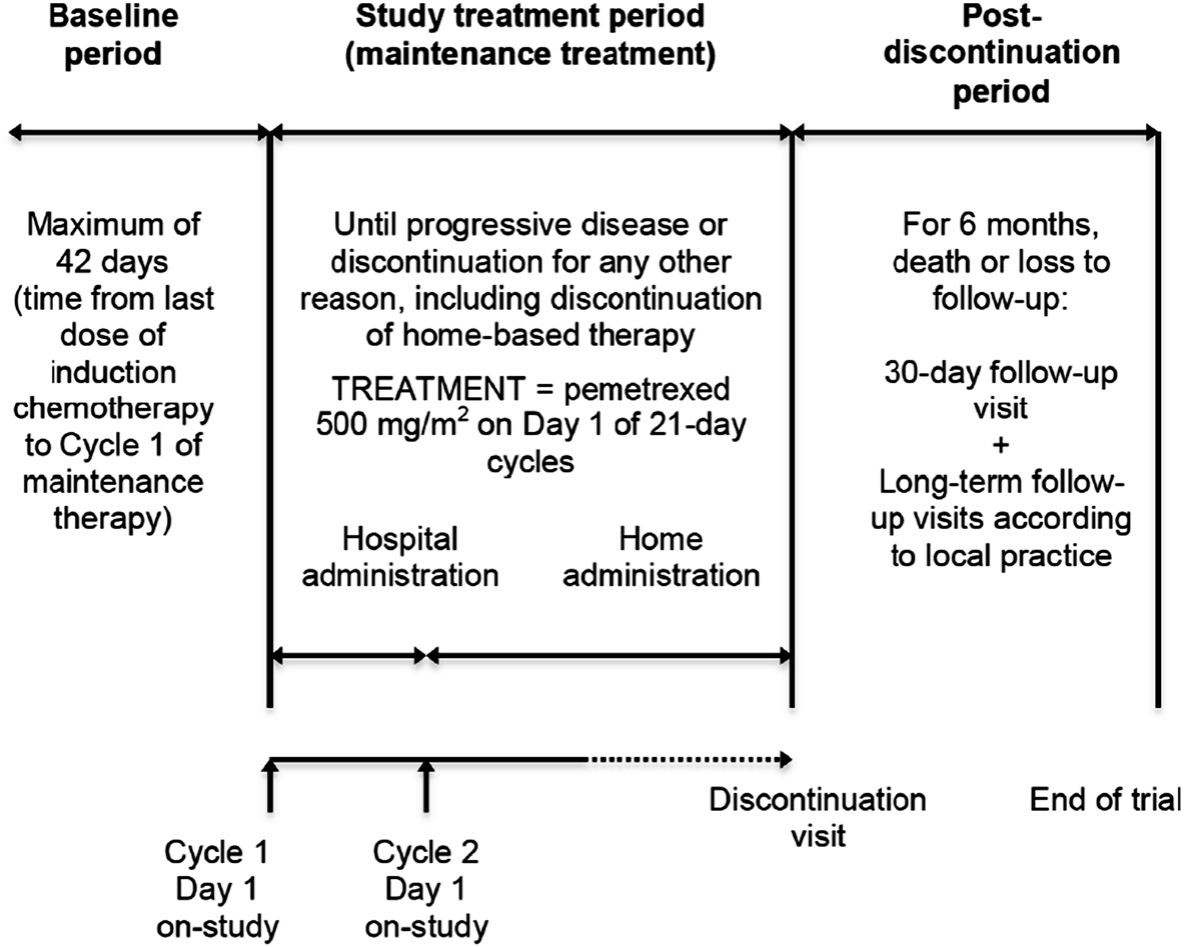
Study design.

The study involves qualified investigative thoracic oncology sites in the UK and Sweden. The sponsor, Eli Lilly, is responsible for overall study management, regulatory affairs, statistical analysis and data quality assurance. The Medical Research Network, an international clinical trial support organisation based in the UK, is providing all support services for the home administration of chemotherapy in this study. All the nurses responsible for home-based chemotherapy administration and care of the enrolled patients are registered nurses who are trained and experienced in administering complex therapies, standard patient assessments and procedures, and recording clinical trial data into source documents or case report forms. The study design is illustrated in Figure 1.

The study protocol has been approved by the ethics review board of each participating institution (investigative site). The study is being conducted in accordance with the ethical principles of the Declaration of Helsinki and Council for International Organizations of Medical Sciences International Ethical Guidelines, the International Conference on Harmonisation Good Clinical Practice Guideline and all applicable laws and regulations. All patients are required to provide a signed informed consent.

### Study population

Patients with advanced (stage IIIB or IV) non-squamous NSCLC that has not progressed radiologically or clinically after four induction cycles of platinum-based doublet therapy (regimen at the discretion of the physician) and who have an ECOG PS of 0 or 1 [19] are eligible for enrolment in this study (Table 1). Tumour responses are assessed objectively using Response Evaluation Criteria in Solid Tumors (RECIST; version 1.1) [20]. Sample size calculations indicated that a minimum of 50 patients is required for this study.

**Table 1:** Study inclusion and exclusion criteria

	
**Inclusion criteria**	Willing to comply with home delivery administration and have family or close environment support willing to comply with home delivery administration
	Histological or cytological diagnosis of non-squamous NSCLC
	Stage IIIB (not amenable to curative treatment) or IV NSCLC prior to induction therapy [21]
	Completed and not progressed after four induction cycles of platinum-based doublet therapy (type at the discretion of the physician). Documented radiographic evidence of a tumour response must occur at the end of Cycle 4 of induction therapy within 3 weeks before receiving the first cycle of study drug (RECIST version 1.1) [20]
	Received on-study treatment no earlier than 21 days and no later than 42 days from Cycle 4 Day 1 of induction therapy
	ECOG performance status 0 or 1 [19]
	Prior radiation therapy is allowed if to <25% of the bone marrow (but not whole pelvis radiation) and the patient has recovered from all toxicities (except for alopecia) before enrolment
	Adequate organ function (bone marrow reserve, hepatic, renal)
	Aged ≥18 years at time of screening
	Females must be surgically sterile, postmenopausal or must have a negative serum or urine pregnancy test within 7 days prior to the first dose of study drug
	Males and females with reproductive potential must agree to use a reliable method of birth control during the study and for 6 months following the last dose of study drug
	Estimated life expectancy ≥12 weeks
	Signed informed consent before any study-specific procedures
**Exclusion criteria**	Squamous cell NSCLC
	Current or prior (within last 30 days) participation in a clinical trial involving an investigational product or non-approved use of a drug or device
	Serious concomitant systemic disorder that according to the investigator, would compromise ability to adhere to the protocol
	Serious cardiac condition, such as myocardial infarction within 6 months, angina or heart disease (NYHA class III/IV)
	Central nervous system malignancy or metastases (screening not required) unless patient is asymptomatic and radiographically stable after local therapy and has been off corticosteroids and/or anticonvulsants for ≥1 week
	Concurrent administration of any other anti-tumour therapy
	Second primary malignancy that may affect interpretation of results
	Inability to interrupt aspirin or other non-steroidal anti-inflammatory agents, other than aspirin ≤1.3 g/day, for a 5-day period (8-day period for long-acting agents)
	Inability or unwillingness to take folic acid or vitamin B12 supplementation, or corticosteroids
	Pregnant or lactating
	Recent (within 30 days) or concurrent yellow fever vaccination

NSCLC = non-small cell lung cancer; RECIST = Response Evaluation Criteria in Solid Tumors; ECOG = Eastern Cooperative Oncology Group; NYHA = New York Heart Association Class.

### Study treatment definition and schedule

Patients must start on-study treatment no earlier than 21 days and no later than 42 days from Cycle 4 Day 1 of induction therapy. Patients receive maintenance therapy with i.v. pemetrexed 500 mg/m^2^, administered over 10 minutes, on Day 1 of a 21-day cycle until progressive disease (PD) or unacceptable toxicity, or until the patient discontinues from the study for any other reason. Pemetrexed is given at the full starting dose to all patients, irrespective of the previous drug regimen or dose given during the induction phase (Figure 1).

For the purpose of this study, study treatment is defined as pemetrexed maintenance therapy received at the hospital (Cycle 1) and, thereafter, any doses received in a home setting. All patients receive folic acid and vitamin B12 supplementation, and prophylactic dexamethasone according to the pemetrexed label.

### Discontinuation from home-based treatment or from the study

Patients are discontinued from the study if they revert to hospital administration of pemetrexed maintenance therapy or when it is decided that home-based administration of pemetrexed should be stopped. Reasons for discontinuation because of the home setting include patient or family decision (e.g. anxiety or dissatisfaction with the home delivery service), physician decision (e.g. complications related to drug administration, such as difficult vein access), or patient non-compliance with study home administration procedures. Hospitalisations for serious adverse events (SAEs) are not considered a reversion to hospital therapy when the patient returns home to continue chemotherapy after the SAE has resolved. In such a situation, the next infusion can be delayed up to 42 days from the beginning of the previous cycle to allow the patient to recover from the SAE and continue pemetrexed maintenance therapy administration at home.

Reasons for patient discontinuation other than because of the home setting include the occurrence of clinical or objective PD; unacceptable toxicity; and investigator/physician or sponsor discretion for reasons other than those relating to the home setting.

### Study and home administration procedures

Physical examination, weight (using the patient’s bathroom scale for all cycles administered in the home), vital signs, ECOG PS evaluation, serum chemistry and haematology laboratory tests, a record of all concomitant medications and completed HRQoL questionnaires are obtained during the baseline period, before the first dose of maintenance therapy, at each cycle during the study treatment period and at the 30-day post-discontinuation follow-up. Tumour assessments and adverse events (AEs) data are collected during treatment and the long-term follow-up period. Baseline procedures and administration of the first cycle of pemetrexed occur at the hospital (the investigative site). After the first infusion (Cycle 1), the investigative site completes a subject referral form referring the patient to home care, confirming that the patient had consented to the referral (informed consent occurred during the baseline period before any study procedures were performed), and containing information required by the home care team for the appropriate scheduling of the home visits. An appropriately trained nurse is then appointed to visit the patient.

The first home visit is conducted on Cycle 1, Day 19 of maintenance therapy (±2 days) to collect blood samples prior to administration of pemetrexed for Cycle 2. At this visit, the home care nurse also collects information on AEs, concomitant medications, ECOG PS, weight and physical examinations (including vital signs, blood pressure and pulse), as well as providing HRQoL questionnaires (the EuroQol 5-Dimensional Scale [EQ-5D] [22] and the Lung Cancer Symptom Scale [LCSS] [23]) to be completed by the patient for the next cycle. Blood samples are delivered to the site’s local laboratory by the home care nurse or shipped by courier, if long distance. The investigator prescribes the dose for the next cycle according to the laboratory data and patient safety monitoring.

Home care nurses are trained to manage and describe any AE that may occur at home, including acute reactions, and provide information to the primary investigator who is responsible for using that information to determine the severity and causality of AEs, prescribe concomitant medication to treat the AE and to adjust pemetrexed doses when required. AE severity is assessed using the National Cancer Institute Common Terminology Criteria for Adverse Events (CTCAE), version 4.0 [24]. In addition, the home care nurse immediately telephones the investigative site to report any SAE (according to usual procedures) and refers the patient to the site if required. During treatment, the primary contact for the patient is always the investigator.

Tumour assessment is performed at baseline. Then, at any time during study treatment, the investigator can schedule radiological tests for tumour assessment, according to local practice at the site, and evaluate these tests to determine whether the patient should continue receiving study treatment.

Resource utilisation questions are answered by patients/caregivers after the first infusion is administered at the hospital (first cycle) and by patients/caregivers and home care nurses at home (all home-based administrations) at specified times within each cycle, as outlined in the data collection form (Table 2). Patient satisfaction with home care is assessed, for those patients who receive at least four maintenance cycles, on the day of infusion of Cycle 4 and, for all patients, at the 30-day post-discontinuation visit (Table 3). In addition, at the end of home treatment, physicians will be asked to evaluate overall satisfaction with home care by answering the question, ‘How would you rate your overall satisfaction with the distant management of the patient during chemotherapy at home (very dissatisfied, somewhat dissatisfied, neither satisfied nor dissatisfied, somewhat satisfied, very satisfied)?’.

**Table 2:** Resource utilisation questions, to be answered by patients/caregivers and the home care nurse*

**Questions**	**Answers**
**A) Questions for the patient to be answered after the first infusion (administered at the hospital).**	
1. How long did the whole process take, from the time you arrived to the hospital until the time you left the hospital?	Hours: Minutes:
2a. How long did the whole process take, from the time you left home until the time you got back home again?	Hours: Minutes:
2b. How far did you have to travel from home to the hospital?	Miles/km (depending on country):
2c. If you are travelling by public transport, how much does the return trip to hospital cost you?	Pounds/Swedish crowns (kronor):
**B) Questions for the patient to be answered before receiving the infusion at home at each cycle. The home care nurse can help the patient to answer these questions.**	
1. After your last chemotherapy, did you have any unplanned visits to the Accident and Emergency (A&E) department?	Yes/No/Unknown
If yes, how many?	1 2 3 4 ≥5
2. After your last chemotherapy, did you require any unplanned visits to your specialist (oncologist, pulmonologist, etc.)?	Yes/No/Unknown
If yes, how many?	1 2 3 4 ≥5
3. After your last chemotherapy, did you require any unplanned visits to your general practitioner (GP) or family doctor?	Yes/No/Unknown
If yes, how many?	1 2 3 4 ≥5
4. After your last chemotherapy, did you require any unplanned diagnostic procedures?	Yes/No/Unknown
If yes, which ones?	Brain MRI
	Brain CT scan
	PET scan
	Chest radiography
	Bone scintigraphy
	Other (specify):
**C) Questions for the patient to be answered after receiving the infusion at home at each cycle**	
1. How long did the whole process take, from the time the nurse arrived until the time the nurse left your home?	Hours: Minutes:
**D) Questions for the caregiver to be answered after the infusion at the hospital**	
1. How much additional time have you taken off work or for other duties related to infusion at the hospital?	Days: Hours: Minutes:
**E) Questions for the caregiver to be answered after the infusion at home at each cycle**	
1. How much additional time have you taken off work or for other duties related to infusion at home?	Days: Hours: Minutes:
**F) Questions for the home care nurse**	
1. To be answered after the infusion at home at each cycle	
a. How long did the whole process take, from the time you left the hospital to the time you got to the patient’s home?	Hours: Minutes:
b. How long did the whole process take, from the time you arrived at the patient’s home until the time you left patient’s home?	Hours: Minutes:
2. To be answered only during the first infusion administered at home.	
How far did you have to travel from hospital to the patient’s home?	Miles/km:

*These questions were developed specifically for the study.

**Table 3:** Patient satisfaction with home care*

**Patients should answer each question in Sections A, B, C, and D (questions 1 to 16) on the day of infusion of Cycle 4 after home administration of study treatment (only patients who have not discontinued from study treatment by Cycle 4) and at the 30-day post-discontinuation visit (all subjects).**	
**A) Please evaluate your hospital experience in this trial**	
1. What do you consider advantages of having chemotherapy at hospital? Please specify which ones (choose all that apply):	Support from other patients
	Access to other medical specialists
	Access to more technical services
	Safer in case something goes wrong
	Other (specify):
2. What do you consider disadvantages of having chemotherapy at hospital?	Need to travel
	Having to wait for treatment
Please specify which ones (choose all that apply):	
	Not having a personalised treatment
	Lack of privacy on the ward
	Other (specify):
3. How would you rate your overall satisfaction with chemotherapy at the hospital?	Very dissatisfied
	Somewhat dissatisfied
	Neither satisfied nor dissatisfied
	Somewhat satisfied
	Very satisfied
4. How would you rate your overall satisfaction with the nursing staff during chemotherapy at the hospital?	Very dissatisfied
	Somewhat dissatisfied
	Neither satisfied nor dissatisfied
	Somewhat satisfied
	Very satisfied
**B) Please evaluate your home experience in this trial**	
5. What do you do consider advantages of having chemotherapy at home?	No need to travel
	Not having to wait for treatment
Please specify which ones (choose all that apply):	
	Personalised service
	More privacy
	Other (specify):
6. What do you consider disadvantages of having chemotherapy at home?	Lack of other patients’ support
Please specify which ones (choose all that apply):	
	Extra burden for family/friends
	Safety concerns
	Need to rely on one medical specialist
	Other (specify):
7. How would you rate your overall satisfaction with chemotherapy at home?	Very dissatisfied
	Somewhat dissatisfied
	Neither satisfied nor dissatisfied
	Somewhat satisfied
	Very satisfied
**C) Could you please provide us additional information regarding your home care nurse during your home treatment?**	
8. Was the nurse an easy person to talk to?	Yes/No
9. When the nurse came, did you feel he/she had enough time to do the required things?	Yes/No
10. Do you think the nurse had time to discuss things with you?	Yes/No
11. Did you feel that the nurse knew enough about you and your illness?	Yes/No
12. Were you able to get all the information you wanted about your illness or treatment?	Yes/No/Uncertain
13. Would you say that the nurse gave:	a lot of reassurance and support;
	some reassurance and support;
	hardly any reassurance and support?
14. How would you rate your overall satisfaction with the nursing staff during chemotherapy at home?	Very dissatisfied
	Somewhat dissatisfied
	Neither satisfied nor dissatisfied
	Somewhat satisfied
	Very satisfied
**D) Could you please evaluate your preferences regarding home and/or hospital treatment?**	
15. Do you prefer having your chemotherapy at home or at the hospital, or are you indifferent?	Home/Hospital/Indifferent
16. Would you recommend having chemotherapy at home to someone else in your same situation?	Yes/No/Not sure

*These questions were developed specifically for the study.

#### Preparation, delivery and home administration of pemetrexed

Pemetrexed is reconstituted according to labelling instructions by the site pharmacy and then either collected by the home care nurse or, if long distance, shipped via specialist courier to the patient on the day of treatment. The infusion bag, properly labelled, is transported in a rigid, refrigerated box; every nurse has a spillage kit in his or her car in case of accident. Transportation to, and then administration of pemetrexed at, the patient’s home must occur within 24 hours of reconstitution. The home care nurse administers pemetrexed using a pump to ensure that the drug is infused over 10 minutes. Once pemetrexed administration is completed, the infusion bags are collected and transported in cytotoxic sharps bins by the home care nurses to their base, where they are destroyed according to local regulations (e.g. under Control of Substances Hazardous to Health regulations).

### Study outcomes assessment

#### Home administration adherence

The duration of adherence will be calculated as the time from the first dose at Cycle 1 (hospital administration) until either the last day of the cycle when the decision is made for the patient to revert to pemetrexed hospital administration or the last day of the cycle when the patient discontinues study treatment or the study for any of the reasons related to the home setting specified earlier. From that time, patients who have met these discontinuation criteria will be considered as non-adherent for the study’s primary objective. Reasons for non-adherence will be noted.

#### Efficacy

TTF is defined as the time from Day 1 of Cycle 1 to the date of the first of the following events: discontinuation of pemetrexed due to toxicity, PD or death due to any cause. The duration of OS is defined as the time from Day 1 of Cycle 1 to the date of death from any cause.

#### Safety and treatment exposure

Patient safety, recorded by maximum CTCAE [24] severity grade and seriousness, as well as hospitalisations and transfusions, will be assessed throughout the entire study treatment duration and at the 30-day post-discontinuation period for all patients who receive at least one dose of study drug. Overall exposure to study drug and any treatment adjustments will be recorded for the entire treatment period.

#### Health-related quality of life

During the baseline period, on Day 1 of each cycle and at the 30-day post-discontinuation visit, patient-reported general HRQoL will be measured using the EQ-5D [22]; disease-related symptoms will be measured at the same time-points using the LCSS (both patient and observer scales) [23].

#### Patient and physician satisfaction

Patient satisfaction with home care, as well as physician/investigator satisfaction with distant management of the patient, will be documented using questions composed for the purpose of this study, at the 30-day post-discontinuation visit and on the day of infusion for Cycle 4, for those patients who receive at least four maintenance cycles.

#### Resource use

The use of healthcare resources (primary care, emergency facilities, hospitalisations, and unplanned diagnostic tests), the number and length of visits, and distances travelled to provide/receive the study treatment will be documented by clinical staff and patients/caregivers, and then collated. As the prices of resources used during the study vary between countries, country-specific costs obtained from external sources will be applied to the resources utilised.

### Statistical considerations and sample size estimation

The primary outcome measure is adherence to treatment administration at home, and the range of the 95% confidence interval (CI) around this estimate was used as the basis for the sample size calculation. Kaplan–Meier survival analysis methodology will be used to estimate the adherence rate as this will account for those patients who are censored (i.e. who die or discontinue treatment and exit the study without prior reversion to hospital administration).

As no information regarding adherence using our definition was available, sample size calculation was based on several data sources. A study using gemcitabine home maintenance therapy found that 1 of 24 patients reverted to hospital infusion [9]; however, to be conservative, we have assumed higher rates for non-adherence. The provisional rates for censoring were taken from the pivotal study of pemetrexed maintenance therapy [17]. Under pre-specified assumptions, and based on Greenwood’s formula for standard error of survival [25], with a sample of 50 patients in the study, the rate of adherence will be 86% by the beginning of the sixth cycle when more than 50% of patients will be expected to discontinue pemetrexed treatment.

For the primary endpoint, the rates of adherence to home administration will be calculated for each cycle and will be reported with their corresponding 95% CIs.

OS and TTF will be estimated using Kaplan–Meier methodology and reported with corresponding 95% CIs. Patient satisfaction and physician/investigator satisfaction will be summarised.

Resource utilisation and HRQoL data will be summarised separately for infusions received at the hospital and at home.

## Discussion

This study aims to answer a number of questions regarding the home-based administration of pemetrexed maintenance therapy and the experience of patients with advanced non-squamous NSCLC. The primary objective is to assess whether home administration of cytotoxic chemotherapy (pemetrexed) is feasible for patients and healthcare personnel, as measured by the adherence rate. Between December 1, 2011 and October 29, 2012, we enrolled 52 patients at nine sites (two in Sweden and seven in the UK).

The Department of Health Cancer Reform Strategy in the UK indicates that care should be delivered in the appropriate setting and that “there are significant opportunities to shift some services from inpatient to ambulatory care… this shift improves patient experience and outcomes and increases the efficiency of services” [8]. In addition, home care may offer benefits to the patient and caregivers, personally, psychologically and socially. In the UK, home chemotherapy is delivered by a few National Health Service (NHS) trusts and by several private healthcare companies to a limited number of patients meeting criteria specific to each organisation [26], and has not been well studied or utilised in that country. However, the recent report ‘Chemotherapy Services in the Community – A Guide for PCTs’ cites evidence from countries such as France and the USA, where home chemotherapy is more frequently used, and Australia and Spain, where home administration of chemotherapy has been studied. The evidence demonstrates that home-based chemotherapy is generally preferred by patients and carers, and has similar or slightly higher costs than hospital-delivered chemotherapy [26].

Our study is being conducted in the UK and Sweden because these European countries allow chemotherapy to be delivered to patients at home by qualified nurses trained in such procedures. UK NHS trusts that are offering home-based administration of chemotherapy provide institutional guidelines for the management of cytotoxic agents that are in general agreement with the protocol of our study. In Sweden, chemotherapy is usually administered only at highly specialised centres; however, these hospitals often service wide geographic areas (in some instances, the distance from the most rural areas to the hospital can be more than 130 km). Therefore, most counties in Sweden have well-developed systems for delivering home care to patients, although administration of chemotherapy is unusual and is controlled by the Swedish Work Environment Authority statute AFS 2005:5.

However, regulations concerning the home administration of chemotherapy differ across Europe, and in a number of countries (e.g. Germany, Italy and Spain), regulatory authorities have provided no specific guidelines regarding home administration of cytotoxic chemotherapy, although some guidance is available. In Italy, home administration of i.v. cytotoxic drugs is not allowed, except in exceptional cases, such as when it is physically impossible for the patient to come to the hospital; in these instances, only a physician can administer home treatment. Similarly, home administration of i.v. cytotoxic drugs is not allowed in Spain or in Germany where the high density of hospitals and oncological/pneumological specialist offices seems to obviate the need for such a treatment strategy. In addition, the i.v. administration of chemotherapeutics is the responsibility of physicians in Germany and cannot be delegated.

In contrast, home administration of selected cytotoxic chemotherapy is allowed and encouraged in France [27], although it appears to be underutilised [28,29], and this country has well-defined official guidelines for such procedures that are in general agreement with the protocols used in our study. In France, in common with guidelines in the UK and Sweden, it is possible for nurses, qualified and trained in the handling, administration and management of cytotoxic drugs, to administer these agents in the home setting. However, in France, the organisation of home care must be co-ordinated and supervised by a physician (with a mandatory patient pre-treatment visit by a physician), and should actively involve the referent general practitioner, who must accept this responsibility [30]. In addition, patients are carefully selected for eligibility, and a protocol of emergency procedures needs to be established and validated by all those involved in the provision of home administration of i.v. cytotoxic chemotherapy, including the patient and caregiver(s). Although the first infusion is required at hospital, some agents are not considered suitable for home administration in France; only cytotoxic agents with easy and safe administration procedures are eligible, and central venous access using a Port-A-Cath device is mandatory for all i.v. drugs administered at home. Agents requiring complex monitoring or those associated with acute and hypersensitivity reactions, such as taxanes, bleomycin, ifosfamide, cyclophosphamide, methotrexate, irinotecan, oxaliplatin and etoposide, are not recommended for home administration in France.

Regardless of country-specific recommendations, it appears that home administration of chemotherapy is not right for all patients and care givers, or for all types of chemotherapy [26,30]. In addition, ensuring that care is safe and effective is essential when considering the place of home chemotherapy within any cancer service [26,27,30].

We selected pemetrexed for this study because it has several features that make it suitable for home-based administration as maintenance therapy in patients with advanced NSCLC. In both registration studies, pemetrexed maintenance therapy was well tolerated and reported AEs were consistent with the known safety profile of the drug [31]. Notably, there was no significant difference in drug-related grade 3 or 4 toxic effects in patients receiving >6 cycles of therapy and patients receiving ≤6 cycles of pemetrexed [17,18], although neutropenia was numerically more common in one study (incidence of 9% in patients receiving >6 cycles versus 4% in patients receiving ≤6 cycles; p = 0.062) [18]. Additionally, these studies found that pemetrexed maintenance therapy had no overall detrimental effect on patients’ quality of life [32,33]. The efficacy of pemetrexed in NSCLC, together with its favourable and well-characterised safety profile [31] and ease of administration (as a 10-minute i.v. infusion) make this drug an appropriate and suitable candidate for administration in an outpatient, home-based setting.

We identified a number of studies that evaluated the feasibility, safety and clinical efficacy of home cytotoxic chemotherapy, several in comparison with chemotherapy delivered in the hospital or outpatient setting [9,11-13,34-45]. However, the results of these studies must be considered in light of the fact that only three were randomised controlled trials [11-13], one of which had high withdrawal rates [12], and many studies were small and provided limited data. In these studies, adult patients with (most commonly) advanced colorectal, breast, head and neck, or gynaecological cancer or bone metastases received short i.v. infusions or bolus injections of cytotoxic chemotherapy. A systematic review of these studies showed that home administration of cytotoxic chemotherapy was associated with similar rates of AEs (overall and severe) as were found in the hospital setting, and that permanent withdrawal of patients from home administration because of AEs or device-related complications occurred infrequently [10]. Although none of the trials was powered to detect differences in efficacy, no evidence was found to suggest that there would be a difference in efficacy of chemotherapy according to treatment administration location. With respect to the acceptability of home-based administration of cytotoxic chemotherapy, patients were at least as satisfied with this location of treatment as they were with outpatient or hospital-administered chemotherapy [10], and evidence from randomised controlled trials [12,13] suggests that HRQoL following cytotoxic chemotherapy was similar in both the home and hospital setting, and that patients may prefer home administration [11,12]. Factors contributing to patient satisfaction with home-based treatment included personalised care from nursing staff, reduced difficulties with transport to hospital, fewer financial concerns, less anxiety, and less disruption to daily and family life.

We identified only one study of direct relevance to the home-based administration of i.v. cytotoxic chemotherapy in patients with NSCLC. That study evaluated the feasibility of home-based administration of i.v. gemcitabine monotherapy for up to 6 months in chemo-naive patients with advanced NSCLC and a PS of 0–2 [9]. In common with our study design, patients received the first cycle of chemotherapy in hospital and subsequent cycles at home. The primary endpoint was the feasibility of home administration, defined as a <25% reversion to hospital care. Investigators found home-based administration of chemotherapy to be feasible and acceptable to patients, with only 1 of 24 patients requiring gemcitabine to be changed from home to hospital administration [9]. Of 13 patients interviewed at the end of Cycle 2 of therapy, all expressed a preference for home-based therapy for reasons that included lower levels of stress, no travel, less waiting around for treatment and more time at home to carry out usual activities. Of the 13 informal carers interviewed, 12 preferred home-based therapy.

The practicality of home administration of cytotoxic agents is an important issue that we address in our study protocol. There are a number of logistic challenges to the provision of an efficient and effective home-based chemotherapy care service. One is the challenge of providing treatment to patients at varying distances from the hospital, particularly in Sweden – this study aims to determine the feasibility both for patients in urban settings and those who live many miles from hospital. One way that we plan to alleviate some of the travelling required by home care nurses will involve the use of a specialist courier to deliver the drug to patients who live a distance away from the site. In the study by Anderson et al. [9], the distance travelled by home-based nurses when visiting a patient ranged from 33 to 113 miles (mean 51 miles). Although these distances did not include travel to the hospital to pick up the chemotherapy, the authors concluded that transferring the administration of single-agent gemcitabine from the hospital to the patients’ homes was a feasible option that did not appear to increase the burden on community services and warranted further investigation [9].

Another challenge to be overcome is the reconstitution of the drug prior to use on the day of infusion. In our study, this is to be carried out by the site pharmacy prior to being picked up by the home care nurse or specialist courier and transported under refrigerated conditions within 24 hours of reconstitution, taking into account the stability properties of pemetrexed.

Because patients will not be seen by their physician before each cycle of maintenance chemotherapy, the quality of nursing staff will be paramount when administering the study drug, collecting laboratory required samples and conducting patient assessments as per the protocol. Home care nurses in this study are fully qualified, trained and experienced nurses and are responsible for recognising, reporting and managing (in the first instance) any serious AEs. Any future home-based care programme of this nature will require the availability of similarly trained personnel to assure the safety of the patient. Patients’ and investigators’ satisfaction with the quality of the home nurse staff, home care service and the distant management of patients is also to be assessed during the study. Due to the general lack of well-accepted standard questionnaires in this setting, satisfaction with home treatment and resource utilisation will be assessed using a set of study-specific questions, developed based on data from literature reviews and elaborated by the study team. The assessment of resource use outcomes is particularly relevant in patients with advanced NSCLC, as the management of this patient group requires a multidisciplinary approach, involving a range of medical specialities, including those managing smoking and age-related co-morbidities.

## Conclusion

In oncology, home-based care is generally reserved for the late palliative setting. This has led to a lack of investigations and data addressing home-based delivery of cytotoxic chemotherapy. Regulations regarding home delivery of cytotoxics differ across Europe, with a notable lack of practice guidelines in most countries, except in France. The feasibility study discussed here will address an important aspect of maintenance therapy in NSCLC – patient comfort during protracted home-based cytotoxic chemotherapy. The study design requires unusual methodology and specific logistics to address outcomes relevant to the home-delivery approach related to home delivery feasibility. Thus, pending the study results, this article presents a study design that offers a novel and reproducible model for home-based chemotherapy, and discusses the practicalities of such an approach.

## Abbreviations

AE: Adverse event; CI: Confidence interval; CTCAE: Common Terminology Criteria for Adverse Events; ECOG: Eastern Cooperative Oncology Group; EQ-5D: EuroQol 5-Dimensional Scale; HRQoL: Health-related quality of life; i.v.: Intravenous; LCSS: Lung Cancer Symptom Scale; NHS: National Health Service; NSCLC: Non-small cell lung cancer; OS: Overall survival; PS: Performance status; PD: Progressive disease; RECIST: Response Evaluation Criteria in Solid Tumors; SAE: Serious adverse event; TTF: Time-to-treatment failure.

## Competing interests

The study was supported by Eli Lilly and Company. Susana Barriga, Nawel Bourayou, Yulia D’yachkova, Maria Lorenzo and Carla Visseren-Grul are all employees of Eli Lilly and Company Limited. Carla Visseren-Grul is a holder of Eli Lilly shares.

Gunnar Hillerdal has no competing interests.

Rohit Lal has received one unrestricted donation of £9,000 from Eli Lilly in the past five years. He has received one restricted grant of £4,000 from Pierre Favre. Neither of these funds relate to this study protocol. He has received one unrestricted donation from Eli Lilly as disclosed above which holds the patent for the IMP relating to the content of this manuscript. He has no stock or shares, or patents pending, and no non-financial interests.

Marianne Nicolson has received honoraria from Eli Lilly for advisory board work and lectures, funding for meeting support and research support. She has no stock or shares, or patents pending, and no non-financial interests.

Anders Vikstrom has received honoraria from Eli Lilly for lectures and has also been a member of an advisory board. He has no stock or shares, or patents pending, and no non-financial interests.

## Authors’ contributions

All authors contributed to the study conception and design, were involved in drafting the manuscript and have read and approved the final version.
